# Covalent Carbide Interconnects Enable Robust Interfaces and Thin SEI for Graphite Anode Stability under Extreme Fast Charging

**DOI:** 10.1002/advs.202408277

**Published:** 2024-10-23

**Authors:** Yverick Rangom, Oleksii Sherepenko, Ahad Shafiee, Alek Cholewinski, Kiran Gundegowda Kalligowdanadoddi, Bersu Bastug Azer, Parisa Jafarzadeh, Boxin Zhao, Elliot Biro, Holger Kleinke, Michael A. Pope

**Affiliations:** ^1^ Department of Chemical Engineering University of Waterloo 200 University Avenue Waterloo N2L3G1 Canada; ^2^ Department of Mechanical and Mechatronics Engineering University of Waterloo 200 University Avenue Waterloo N2L3G1 Canada; ^3^ Department of Chemistry University of Waterloo 200 University Avenue Waterloo N2L3G1 Canada

**Keywords:** carbide interconnects, extended cycle life, extreme fast charging (XFC), graphite, Li‐ion batteries, solid electrolyte interface (SEI), titanium hydride

## Abstract

Carbonaceous and carbon‐coated electrodes are ubiquitous in electrochemical energy storage and conversion technologies due to their electrochemical stability, lightweight nature, and relatively low cost. However, traditional reliance on conductive additives and binders leads to impermanent electrical pathways. Here, a general approach is presented to fabricate robust electrodes with a progressive failure mechanism by introducing carbide‐based interconnects grown via carbothermal conversion of (5 wt%) titanium hydride nanoparticles. This method concurrently enhances both electrical and mechanical properties within the electrode architecture. The resulting chemical bonding between active materials establishes a novel mechanism to maintain stable electrical pathways during cycling. Employed as Li‐ion battery anodes, these electrodes exhibit improved cyclability, achieving 80% capacity retention after 800 fast‐charge cycles at moderate loading (1 mAh cm^−^^2^). High loading cells with areal capacity of 3 mAh cm^−2^ show significantly improved cycle life over the same number of cycles. This performance improvement is attributed to the absence of significant impedance growth and a thinner solid electrolyte interphase (SEI) layer formed at high current densities (4C) as demonstrated by X‐ray photoelectron spectroscopy and transmission electron microscopy studies. The enhanced conductivity facilitates SEI formation, lowering ionic impedance and mitigating lithium plating, ultimately leading to the reported extended cycle life.

## Graphical abstract

Extreme fast charging (XFC) of Li‐ion batteries is limited by poor anode conductivity, causing lithium plating and degradation. This study introduces covalent carbide interconnects that bond anode particles to each other and the current collector, enhancing conductivity and mechanical strength. This allows for a thinner, more ionically conductive solid electrolyte interface, enabling repeated XFC with minimal degradation.

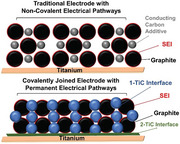

## 1 Introduction

Electrode architectures have seldom changed since the early days of Li‐ion batteries (LiBs). Commercial electrodes almost exclusively use a slurry‐based architecture with polymer as binder and a percolated network of carbon additive to ensure electron conduction throughout the electrode. While scalable and low cost, this architecture ensures neither optimum electron conduction nor permanent electrical pathways. As a result, local overheating, binder degradation, and electrical impedance growth over time degrade particle–particle interfaces and/or electrode‐current collector interfaces over cycling.^[^ ^1^, ^2^, ^3^ ^]^ Impedance growth comes from disruption of electrical and ion channels. At the surface of particles, continuous growth of solid electrolyte interphase (SEI) disrupts both ion and electrical transport by building an insulating layer on the surface of particles.^[^ ^4^ ^]^ Mechanical loss of electrical contact mainly, between current collector and film (delamination) is a major factor in impedance growth.^[^ ^5^ ^]^ Improving and maintaining electrical conductivity can be achieved by incorporating high aspect ratio conductive additives like conductive polymers,^[^ ^6^, ^7^, ^8^, ^9^ ^]^ graphene‐derivatives,^[^ ^10^, ^11^, ^12^ ^]^ MXenes, carbon nanotubes (CNTs), etc.^[^ ^13^, ^14^ ^]^ These materials lower the percolation threshold, improve network conductivity, and, in some cases, increase the mechanical strength.^[^ ^15^ ^]^ Another approach is through 3D current collectors whose biggest appeal is their ability to reduce the thickness of the active material while maintaining loading. Reduced thickness has the advantage of reducing cracking of the film and it directly improves the electrical conductivity by reducing the distance electrons have to travel to the current collector.^[^ ^16^, ^17^ ^]^ At equivalent loading, hierarchically designed 3D current collectors also can improve ion transport by improving the geometry of ion channels.^[^ ^18^, ^19^ ^]^ Unfortunately, these high surface area additives typically increase irreversible capacity loss and can become electrically isolated since they are not strongly bound to the active material.^[^ ^20^ ^]^ Alternatively, monolithic, 3D current collectors made from metal foams, etched metal foils, carbon foams, etc. can form permanent electrical percolation throughout the electrode and can enable high loading electrodes.^[^ ^13^, ^21^, ^22^, ^23^, ^24^, ^25^ ^]^ However, these often add significant weight to the electrodes and are difficult to fill with active materials, reducing scalability and leaving significant dead weight and volume. Furthermore, in most cases, the current collector does not form strong bonds with the active material.^[^ ^26^ ^]^ More recently, the concept of an integrated, monolithic electrode has been established which uses various dealloying approaches to selectively leach away components from a metal or metal oxide film.^[^ ^27^, ^28^, ^29^, ^30^ ^]^ While promising results have been achieved, such approaches are limited to cathode/anode materials composed of certain types of metal/metal oxides.

**Figure 1:**
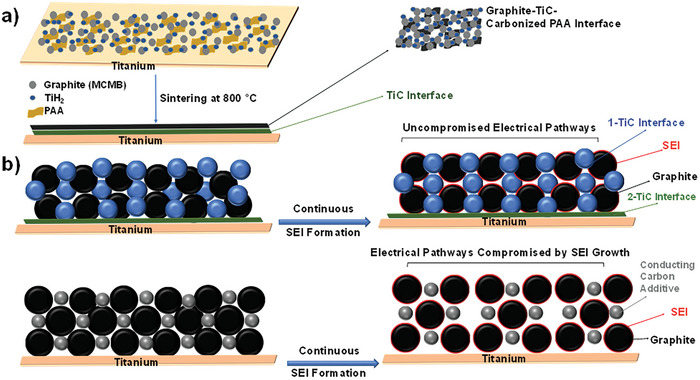
Schematic and proposed mechanism for continuous integrity of electronic pathways as SEI grows. a) Schematic illustration of the sintering process of graphite electrode with titanium dihydride as titanium carbide (TiC) precursor. b) Schematic of retention of electronic pathways with permanent covalent TiC joint versus loss of electrical contact due to SEI growth in traditional slurries with conductive additive particles.

With the aim of designing a similar approach but for slurry‐cast, carbon‐based or carbon‐coated electrodes, this work explores the use of carbide chemistry to join adjacent particles in the electrode network to each other and to the current collector. Full conversion of carbon to titanium carbide (TiC) is known to occur upon heating of carbon blacks with titanium hydride.^[^ ^31^ ^]^ This and similar carbothermal reactions are already commercial production routes to transition metal and carbide powders. Instead of full conversion, as shown schematically in **Figure** **1**a,b, we aim to convert the carbon only partially to TiC at the points of contact with the titanium hydride powder. Since TiC is an electronic conductor and exhibits high electrochemical stability, the resulting carbide‐joined architecture has the potential to form a more permanent network for electrical and mechanical percolation. The use of titanium hydride (TiH_2_) powder also offers the possibility of partially alloying with metallic current collectors like titanium and copper metal which is also expected to lower impedance and improve adhesion.

Such an architecture is of significant interest when considering the failure modes associated with extreme fast charging (XFC, 15 min charge to 80% capacity) of Li‐ion batteries. According to the US Department of Energy (US DoE) fast charging is a critical requirement for mass adoption of electric vehicles (EVs) and durability under repeated fast charges is required for establishing a used EV market.^[^ ^2^, ^9^, ^32^, ^33^, ^34^, ^35^, ^36^, ^37^, ^38^, ^39^, ^40^ ^]^ The 1.32 trillion USD used car market representing 24% of all car purchases worldwide in 2023.^[^ ^41^, ^42^ ^]^ Durability under frequent fast charging routines remains a significant technological challenge.^[^ ^43^, ^44^, ^45^, ^46^ ^]^ The causes for loss of cycle‐life in LiBs are numerous. They include metal plating,^[^ ^47^, ^48^ ^]^ continuous solid electrolyte interphase and impedance growth,^[^ ^4^, ^49^, ^50^ ^]^ degradations due to overheating such as binder degradation causing delamination,^[^ ^2^, ^32^ ^]^ electrolyte decomposition,^[^ ^51^ ^]^ and cathode material fragmentation.^[^ ^35^, ^52^ ^]^ With all but two of these phenomena affecting the anode, it is widely understood that the anode is the limiting electrode especially under fast charging applications.^[^ ^53^, ^54^ ^]^ These limitations are exacerbated due to the high current densities of fast charging combined with the low average electrode voltage that is near 0 V versus Li/Li^+^.^[^ ^55^, ^56^ ^]^ It is also worth noting that unlike the particle fragmentation mechanism occurring at the cathode, anode degradation is largely related to failure of the electrode architecture: i) Mechanical failure of interfaces between particles and current collector (delamination and formation of electrically isolated material) due to binder degradation. Progressive physical separation of materials directly causes impedance growth, but it also allows for new SEI formation to grow between particles further reducing electrical conductivity; and ii) limited charge transport capability for electrons leading to a sustained potential gradient combined with ion transport at the particle to electrolyte interface causing uneven ion depletion and leading to lithium plating at the anode surface. While the covalently joined architecture preserves mechanical integrity and electronic pathways, it also significantly increases electrical conductivity compared to traditional electrodes with percolated conductive networks.

Improved electrical conductivity in an anode opens the door for high current density formation protocols for the SEI that could not be implemented previously.^[^ ^57^, ^58^ ^]^ In our previous work, we demonstrated that performing formation steps under high current densities (> 1C instead of < 0.1 C) using electrodes with high electrical conductivity allows for SEI layers with significantly higher ionic conductivity. This accelerates Li‐ion transport through the graphite particles, helping to prevent lithium plating—a necessary step to enable repeated XFC protocols.^[^ ^59^, ^60^ ^]^ High rate SEI formation can also reduce the cost of LiBs by 6% while increasing manufacturing throughput by reducing formation time from days to hours.^[^ ^58^, ^61^, ^62^, ^63^ ^]^

In the following, we first demonstrate the ability to form TiC interconnects between commercially sourced graphite particles in a LiB anode and to partially bond the electrode to metallic current collectors. The electrical and mechanical properties of the electrodes and their adhesion to both titanium and copper current collectors are assessed to prove the concept. Then, electrodes, containing only a small amount of titanium precursor (<5 wt%) are tested as LiB anodes for cycle‐life under repeated XFC charging. Compared to controls that rapidly fail under these conditions, the TiC‐joined architecture demonstrates negligible capacity fade over 800 cycles with effectively no impedance increase.

## 2 Results and Discussion

**Figure 2:**
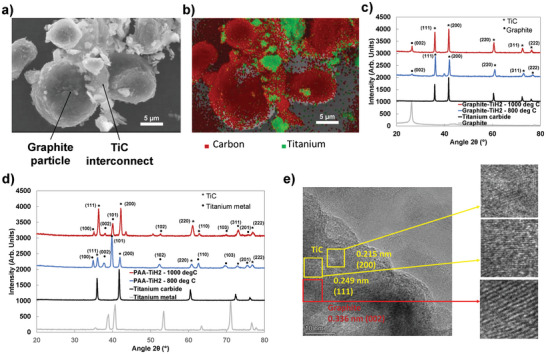
Chemical and morphological characterization of particle‐to‐particle covalent joint. a) Secondary emission SEM image of two graphite particles joined with titanium carbide interconnect at 5 kX magnification. b) Corresponding EDS mapping of carbon and titanium atoms—accelerating voltage 20 kV. c) X‐ray diffraction patterns for graphite‐TiH_2_ mixture (4:1 molar ratio) sintered at 800 and 1000 °C and for non‐sintered titanium carbide and graphite powders. d) X‐ray diffraction patterns for poly‐acrylic acid and TiH_2_ mixture (1:1 mass ratio) sintered at 800 and 1000 °C and for non‐sintered titanium carbide powder and titanium metal. e) TEM imaging of continuous medium of graphite–TiH_2_ mixture (4:1) sintered at 1000 °C.

The TiC interconnects formed through the sintering of TiH_2_ particles in close contact with graphite particles are demonstrated using scanning electronic microscopy (SEM) and corresponding energy dispersive X‐ray spectroscopy (EDS) mapping as shown in **Figure** **2**a,b, respectively. Figure 2a shows the spherical mesoporous carbon microbeads (MCMBs) that are easily distinguished from the smaller, sharp‐edged titanium precursor. EDS mapping confirms the elemental distribution of carbon and titanium where, after sintering, particles that have a similar shape to the titanium precursor, now contain titanium and carbon. In similar carbothermal conversion reactions, the carbon is known to have a higher mobility than titanium in the solid state and diffuses to react with TiH_2_ to form TiC.^[^ ^64^, ^65^ ^]^ Powder X‐ray diffraction (XRD) on these samples indicates a mixed phase of graphite and TiC and the absence of any crystalline phases corresponding to the TiH_2_ precursor when the sintering temperature is 1000 °C. The success of this process was dependent on the proximity of the TiH_2_ to the graphite particles. As shown in Figure S2a (Supporting Information), precursor mixtures that are not compacted prior to sintering do not form TiC even if the powders were ball‐milled. This demonstrates that carbon atom migration is exclusively in the solid state between closely contacting particles. For other carbon precursors like Ketjen black (KB), the joining reaction occurs under sintering temperature as low as 400 °C for incomplete conversion and 600 °C for complete conversion (cf. Figure S2b, Supporting Information). This suggests that the reaction is dependent on the surface area and relative stability of the carbon precursor. For this system, complete conversion occurs relatively quickly, in less than 10 min (cf. Figure S2c, Supporting Information).

For battery testing, since it was challenging to slurry cast a crack‐free electrode film without a polymer binder, electrodes were cast with polyacrylic acid (PAA), which was carbonized upon the carbothermal conversion (cf. Figure 2d). Thermal gravimetric analysis (TGA) indicated that PAA lost 90% of its mass to decomposition leaving behind a carbonized by‐product (cf. Figure S2, Supporting Information). This carbonized by‐product was also mixed with TiH_2_ (2:1 weight ratio) and heated to 800 and 1000 °C. However, as shown in Figure 2d, the carbothermal reaction was incomplete, leaving a mixture of TiC and titanium metal after sintering. Furthermore, the formation of TiC interconnects with the carbonized PAA binder continues up to 1000 °C (cf. Figure 2d) while it is complete at 800 °C when graphite particles are used (cf. Figure 2c). Therefore, while TiC interconnects can form with residue left from the carbonized binder, the reaction preferentially occurs between graphite particles in the electrode architecture.

High resolution transmission electron microscopy (TEM) was used to study the interface between the graphite and TiC formed. As shown in Figure 2e, there is a seemingly continuous transition from domains with the graphitic spacing to crystalline planes associated with TiC as indicated in the figure. The crystalline structure changes from graphite to TiC in the plane of focus as well as perpendicularly to the plane of focus suggesting the formation of covalent bonding (C─C) or partially ionic bonding (Ti─C) between the two phases.

**Figure 3:**
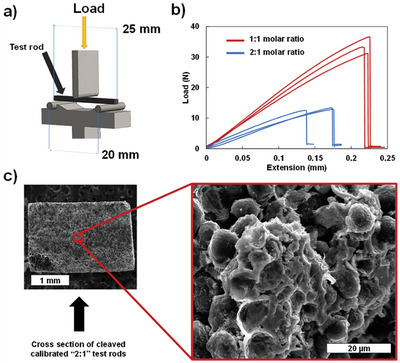
Mechanical properties of graphite–TiC composite material. a) Schematic of ASTM C1161‐18 assembly featuring the position of bench and sample test rod. b) Bending test comparison of 1:1 (red) and 2:1 (blue) molar ratio graphite‐TiH_2_ 25 mm rod samples sintered at 1000 °C. c) SEM images at low (50×) and medium (2 k×) magnification of fractured 2:1 test rod.

To assess the mechanical properties of the composite, mechanical bending tests were performed on the graphite–TiC composite materials following the ASTM C1161 standard method^[^ ^66^ ^]^ for flexural strength of advanced ceramics at ambient temperature using a custom‐made bench as shown in **Figure** **3**a. Such two composites sintered, respectively, from 1:1 and 2:1 molar ratios of graphite to TiH_2_ were tested for bending strength. Additionally, 4:1 molar ratio pellets of sintered mixture were compared to non‐sintered pellets of the same mixture and were used to illustrate the effect of formation of TiC on the mechanical integrity of the composite material. The cold pressed, non‐sintered pellet sustained a crushing pressure of 0.24 MPa while the hot‐pressed, sintered pellet sustained 9.47 MPa before it failed showing 39 times increase in mechanical strength. This large increase indicates the formation of strong interparticle bonds in addition to the van der Waals forces holding the agglomerated cold pressed sample together. From the bending strength test, it is also clear that increasing the amount of the joining TiH_2_ compound increases mechanical strength significantly: from 13 N load for the 2:1 ratio mixture to over 30 N load for the 1:1 ratio (cf. Figure 3b). The SEM imaging of the fractured samples, presented in Figure 3c, reveals that separation occurs at the interface with the graphite particles. This is to be expected as the covalent joint only extends a few nanometers as revealed by the TEM (cf. Figure 2e) characterization combined with the layered morphology of the MCMB graphite material. The fracture image also reveals percolated TiC structures inside the composite material. It is therefore likely that most of the mechanical strength comes from these continuous, bonded, percolated networks.

The new architecture made from sintered (carbonized) polymers and covalent interconnects presents electrical pathways through the network carbonized polymer as well as the interconnects themselves. In terms of electron transport, the proposed architecture outperforms the conductivity plateau of a percolated network of conductive additives as demonstrated by 4‐probe areal conductivity measurements. A traditional slurry made with 5 wt% PAA, 5 wt% super P, and 90 wt% graphite was compared with a sintered slurry made with 50 parts PAA, 5 parts TiH_2_, and 90 parts graphite. After sintering, thermogravimetric analysis shows that 90% of the PAA decomposes into gaseous products leaving approximately 5 wt% carbonized by‐product, 5 wt% TiC, and 90 wt% graphite. The uncompacted traditional graphite slurry film shows a conductivity of 4.47 S cm^−1^ (in‐plane sheet resistance of 25.6 Ω per square). This is consistent with a percolated network of carbon particles, while the sintered graphite film exhibited a measured conductivity of 14.5 S cm^−1^ (in‐plane resistance of 3.3 Ω per square). Since both films were not compacted and the density of the sintered graphite film was lower, due to gas evolution during binder burn‐off, the improvement in conductivity must be related to the reduction in interparticle contact resistance upon covalent joining. The conductivity of the film with covalent joints is therefore 3.24 times higher than the traditional film and most importantly above the maximum conductivity plateau achievable by percolated networks of conductive carbon particles regardless of the amount.^[^ ^67^ ^]^ Traditional percolated networks of carbon black nanoparticles tend to experience a maximum conductivity plateau that occurs quickly after percolation is achieved. For carbon black particles in a polymer matrix, this conductivity plateau is up to 10 S cm^−1^ and adding more carbon black after percolation has been reached has little effect on improving further electrical conductivity.^[^ ^67^, ^68^ ^]^ This is explained as in traditional slurry‐based electrodes, electrons are conducted only by the percolated network of graphite particles and conductive additive as the polymer binder is non‐conductive. In the sintered and covalently joined film architecture, the entire film is electrically conductive, electrons can travel through both the percolated network of graphite particles and the formed TiC material as well as through the sintered/carbonized polymer resulting in the significantly higher conductivity. It is also likely that the direct bonding between carbon and TiC lowers or eliminates the contact resistance between particles at these joints.

**Figure 4:**
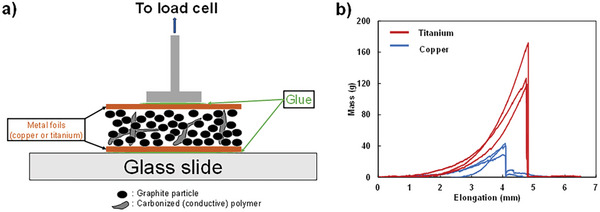
Physical characterization of current collector‐to‐film interface via tear test of graphite, TiH_2_ in PAA slurry films sandwiched between two titanium foils or two copper foils and sintered at 1000 °C. a) Schematic of test rig. b) Test result for titanium (red) and copper (blue) foils.

Applying this approach to create a graphite anode for Li‐ion battery applications, we also surmised that adhesion of the electrode material to the current collector could be improved using a similar approach whereby carbon will react with a titanium current collector to form TiC at the interface between electrode and current collector.^[^ ^69^ ^]^ Titanium current collectors are known to be particularly suited to forming efficient durable conductive bonds with active materials as demonstrated previously by research on direct growth of CoO, Co_3_O_4,_ and Li_4_Ti_5_O_12_. Thanks to their low density of 4.51 g cm^−3^, titanium current collectors are lighter than the copper foil typically used as the anode current collector. Finally, titanium has a higher tensile strength making them easier to handle.^[^ ^70^ ^]^ This system is compared to electrodes sintered on more traditional copper current collectors that do not form carbides. To confirm improved adhesion of electrodes sintered on titanium, tear tests were conducted on symmetrical electrode sandwiches made from sintered slurries of PAA and graphite in‐between two titanium or two copper foils (cf. **Figure** **4**a). Figure 4b shows the result of these tear tests with copper sandwiches supporting only about 40 g of weight while equivalent titanium sandwiches topped 160 g. This shows that there is an active adhesion mechanism with the titanium sandwiches. Interestingly the carbon from sintered PAA diffuses to form TiC bonds with the titanium current collector as characterized by XRD as shown in Figure S2d (Supporting Information). These tests were conducted with the slurry cast electrodes using the same 5 wt% TiH_2_ contrary to the free‐standing rods for mechanical testing that requires a minimum of 25 wt% TiH_2_. On the other hand, graphite particles and titanium foil do not form TiC under the same conditions as shown in Figure S2e (Supporting Information) likely leaving most of the chemical bonding of the film to the titanium current collector to the carbon from the sintered PAA.

### 2.1 Carbide Joined Graphite Anodes Applied to XFC Li‐Ion Batteries

**Figure 5:**
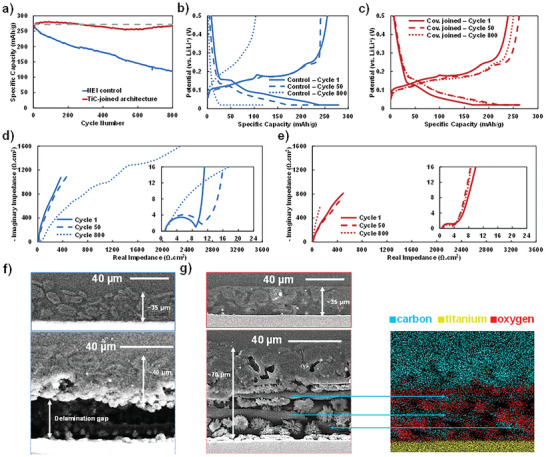
Electrochemical characterization of 1 mAh cm^−2^ graphite anode half‐cells with 4 mAh cm^−2^ NMC‐811 counter electrodes and SEM images of cross sections of the electrodes before and after cycling. a) Cycle life comparison of covalently‐joined architecture versus traditional slurry—15 min charge (3.2C constant current, 0.02 V versus Li/Li^+^ constant voltage) and 1C discharge. Charge–discharge profiles of 1 mAh cm^−2^ graphite anodes after 1, 50, and 800 cycles b) for a traditional slurry architecture electrode c) and for a covalently joined architecture electrode. Electrochemical impedance spectroscopy of 1 mAh cm^−2^ graphite anodes after 1, 50, and 800 cycles d) for a traditional slurry architecture electrode e) and for a covalently joined architecture electrode. f) SEM images of an electrode cross section for the control traditional slurry electrode before cycling (top) and after 800 cycles (bottom). g) SEM images of an electrode cross section for the covalently joined electrode before cycling (top) and after 800 cycling (bottom) with corresponding EDS mapping of carbon, titanium, and oxygen (bottom right).

We now assess how this new electrode architecture with improved cohesion, adhesion, and conductivity can be used to design longer lasting lithium‐ion batteries when subjected to frequent extreme fast charging (XFC) protocols. Thus, we compare this new architecture to commercially fabricated electrodes subjected to an accelerated aging protocol where graphite anodes are repeatedly fast charged for 800 cycles at room temperature. In **Figure** **5**a, initially, both the commercial benchmark and TiC‐joined architecture fulfill the XFC requirement with 80% of the nominal capacity available on discharge. However, the benchmark's performance quickly decays as this electrode experiences continuous capacity loss. On the other hand, the TiC‐joined electrode maintains capacity all the way through the 800 cycles. Both electrodes are made with the same graphite powder, therefore failure of the active material can be ruled out. The electrode architecture is the only difference. Figure 5b,c shows the evolution of the lithiation (charge) curve for the commercial and TiC‐joined architecture electrodes, respectively. The evolution of the curve for the commercial electrode exhibits, first, the collapse of the charge gained through constant current from about 230 mAh g^−1^ during the first cycle to less than 30 mAh g^−1^ at cycle 800. Simultaneously the charge obtained during the constant voltage part of the profile increases from about 45 to 90 mAh g^−1^. Those gains, however, are far from enough to compensate for the losses incurred during the constant current portion of the charge profile. Conversely, the capacity for the covalently joined electrode architecture increases slightly from cycle 1 to 50, most likely due to incomplete initial wetting with electrolyte and then it stays virtually unchanged up to cycle 800. The capacity gained from the constant current and constant voltage are closely matched from cycle 50 to 800 denoting that this novel electrode architecture has not suffered any degradation. To determine the impact that TiC has on the capacity measured for the covalently joined electrodes, pure TiC electrodes were produced by reacting stoichiometric amounts of TiH_2_ and graphite and cycled at the same current densities as the graphite anodes (Figure S10, Supporting Information). At the equivalent current density of a 0.1 C charge, the capacity was 280 mAh g^−1^ while the 3.2C charge only exhibited 7.95 mAh g^−1^. Since only approximately 5wt% of TiC is present in the sintered architecture, it contributes less than 1% of the measured capacity. This suggests its role is solely to improve the network conductivity.

Electrochemical impedance spectroscopy (EIS) analysis was conducted on both electrode types. In this study, EIS is conducted when the graphite electrode reaches 2 V versus Li/Li^+^ to eliminate the impedance contribution from lithium intercalation at the anode site and restricting charge storage of the anode to capacitive storage on the surface of the working electrode. While the charge storage mechanism on the cathode is not restricted to capacitive, in this test storage will also be fully capacitive in nature as this 4 mAh cm^−2^ nickel manganese cobalt oxide (NMC) electrode has a surface area 1.57 larger than that of the 1 mAh cm^−2^ graphite working electrode according to the manufacturer. Therefore, the EIS profiles are reduced to the features found on Nyquist profile for supercapacitors: the trace crosses the real axis at a positive value denoting ionic impedance in the solvent, a semi‐circle denoting the electrical impedance and a large tail denoting the capacitance (height) and ionic impedance due to diffusion phenomena in the electrodes.^[^ ^70^, ^71^ ^]^ An equivalent circuit of this supercapacitor is depicted in Figure S5 (Supporting Information) with R1 representing the series resistance pertaining to solvent resistance to ion transport, R2/CPE2 (where CPE is the constant phase element frequently used to represent an imperfect capacitor with a phase angle less than 90°) representing the global electronic interface, R3/CPE3 representing the global ionic interface, and W representing Warburg‐like capacitance and ion diffusion. This model is used to fit the EIS plot for each cell presented in this work. Fitting of the electronic interface represented on the Nyquist plot by the high frequency semi‐circle close to the x‐axis was prioritized to accurately assess this difference between samples. This emphasis is made by restricting the fitting range to this semi‐circle, obtaining the R2/CPE2 value and then fixing these values as the fitting range is expanded. The cathode is the same in all cases, therefore the evolution of R2 as cycling progresses allows us to follow the electrical impedance of the different graphite anode architectures. The values of R2 for each cell after 1, 50 and 800 cycles are shown in Table S1 (Supporting Information). Figure 5d shows a large increase of the semi‐circle for the control graphite electrode as well as the height of the tail compared to electrode with covalently joined architecture (Figure 5e). The former increase is directly associated with an increase of electrical impedance from 2.9 Ω cm^2^ after 1 cycle to 61.4 Ω cm^2^. This is more than a 20‐fold increase in electrical impedance. At the same time, the height increase denotes a loss of electrically connected surface (i.e., dead active material). Dead active material occurs by permanent separation of active materials either through delamination of the entire film or disintegration of part of the film.^[^ ^72^ ^]^ This measured increase of impedance confirms this well‐known phenomenon.^[^ ^73^, ^74^ ^]^

Delamination of the film from the current collector is confirmed by the cross‐section SEM image of the electrode depicted at the bottom of Figure 5f. Interestingly, and unsurprisingly, in view of the steady electrochemical performance of the covalently joined architecture, the Nyquist plot for this electrode architecture shows only a very small change in the size of the semi‐circle and a decrease of the height of the tail. The decrease of tail height on cycle 800 is likely due to cracking and progressive opening up of the film of the electrode such as cracking in the carbonized polymer exposing more surface area to the electrolyte over time as shown in the cross‐section image at the bottom of Figure 5g. From fitting the Nyquist plot we found a comparatively small increase of electrical impedance from 3.3 to 4.3 Ω cm^2^. This is a confirmation that the intended purpose of having permanent electrical pathways has indeed remained undisturbed including both the particle‐to‐particle and particle‐to‐current collector interfaces. EDS characterization shown at the bottom right of Figure 5g shows that conductive carbon channels remain connected to the current collector for the covalently joined architecture whereas there is a clear empty delamination gap for the control electrode. Contrary to the control electrode from NEI corporation, the TiC‐joined electrode is more robust to repeated strain over time without catastrophic separation or delamination from the current collector or the formation of dead or inactive electrode materials. As shown at the top of Figure 5f,g, the NEI electrode barely changes in thickness starting at about 35 µm and ending at around 40 µm, while the covalently joined electrode also starts with 35 µm thickness that then becomes about 70 µm after the 800 cycles are completed denoting a more forgiving mechanical failure mode compared to the catastrophic mechanical failure mode of the control NEI traditional electrode.

To determine whether the improved cohesion between particles, adhesion to the current collector, or both are responsible for the improved stability, we tested electrodes fabricated with copper current collectors and titanium current collectors with and without TiH_2_ addition prior to sintering. Removing the TiH_2_ precursor from the sintered electrode formulation does not change the good cycle‐life performance as long as the titanium current collector is used (cf. Figure S6a,d, Supporting Information) with small electrical impedance increases from 1.8 to 2.2 Ω cm^2^ after 800 cycles (1.2‐fold increase). It is worth noting that the impedance of this cell without TiH_2_ displays an electrical resistivity that is 1.8 times smaller than the equivalent cell with TiH_2_. While the reason for this is not clear, we can speculate that without TiH_2_ particle more carbon from PAA has formed a more coherent TiC joint with the titanium current collector. Cycle life of this latter cell with titanium current collector is also almost indistinguishable from that of the cell with TiH_2_ and titanium current collector. However, the TiH_2_ precursor was instrumental in controlling the growth of electrical impedance through 800 cycles of equivalent sintered electrodes using copper foils as current collectors (cf. Figure S6b,c,e,f, Supporting Information). Interestingly, the sintered cell without TiH_2_ exhibits electrical impedance 1.5 times larger than the equivalent copper cell with precursor. Since copper cannot form chemical bond with carbon, it seems that the TiH_2_ precursor is providing long lasting electrical pathways with the copper current collector. This suggests two alternative routes to maintaining good electrical contact over many fast‐charging cycles: i) improving adhesion using TiC interconnects to the titanium current collector which provides sufficient electrical percolation pathways from current collector through the tens of microns thick electrode film or ii) improving cohesion between particles via TiC interconnects between graphite particles that can maintain conductivity laterally even if the electrode fails adhesively. Using both routes simultaneously clearly leads to the most robust electrodes with longer cycle life. The use of copper current collectors over titanium ones is preferred from a cost perspective.

Finally, the higher electrical conductivity of the electrode also allows for conducting the SEI formation step under much higher current densities. SEI layers formed at higher rates tend to be thinner and more transparent to ion transport therefore increasing ionic conductivity.^[^ ^59^, ^60^ ^]^ In this study, SEI formation at 4C was paramount in allowing stable cycling as shown in Figure S7b (Supporting Information) while covalently joined electrodes with SEI formed under 0.1C showed a rapidly decreasing capacity in the first 10 cycles followed by continuous capacity loss. Electrodes with SEI formed under 4C show no capacity loss. The control slurry‐based electrode shown in Figure S7a (Supporting Information) showed capacity loss regardless of the SEI formation current. However, the control electrode with SEI formed under 0.1C clearly exhibited signs of lithium plating during the very first fast‐charge cycle as the specific capacity on the charge cycle approached 700 mAh g^−1^ largely, exceeding the theoretical capacity of graphite. This further demonstrates the increased need for less ionically resistive SEI layers formed under higher rate to allow for reliable XFC cycling.

**Figure 6:**
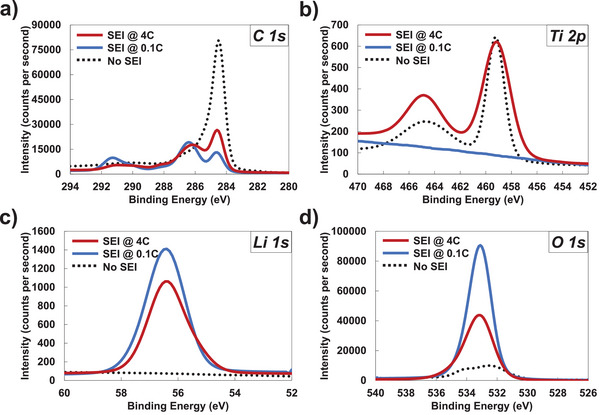
XPS characterization of electrodes with no SEI (pristine), SEI formed @ 0.1C and SEI @ 4C for a) carbon 1s bonds, b) titanium 2p bonds, c) lithium 1s bonds, and d) oxygen 1s bonds.

While it was proven in our previous studies on thin film electrodes with high conductivity that a higher rate formation cycle leads to a thinner and more porous SEI that leads to higher ionic conductivity,^[^ ^59^ ^]^ we wanted to confirm this was the case for the thicker, higher loading TiC‐joined electrodes. To do so, we imaged the SEI layer using high resolution TEM and measured a significantly different thickness of SEI layers (by Student t‐test, 95% confidence interval) formed at 0.1C and 4C on TiC‐joined electrodes which were found to be 5.21 ± 0.45 nm and 3.99 ± 0.66 nm, respectively as shown in Figure S8 (Supporting Information). X‐ray photoelectron spectroscopy (XPS) was also carried out on TiC‐joined electrodes after the SEI formation step was carried out at either 0.1C or 4C and compared to the intensities to uncycled controls. This is a surface‐sensitive technique that only probes the composition within ≈5–10 nm of the surface and the intensity or blocking of intensity from the underlying active material can be a qualitative indicator of SEI thickness. The high‐resolution spectrum for C 1s, Ti 2p, Li 1s, and O 1s are shown in **Figure** **6**. The C 1s signal (Figure 6a) for the uncycled electrode shows the largest intensity peak around 284.6 eV corresponding to sp^2^ hybridized carbon (C═C). In the samples examined after SEI formation, there is a significant reduction in this 284.6 eV peak intensity due to the formation of SEI. The intensity reduction is the largest for 0.1 C suggesting that this is the thickest layer while a thinner SEI layer resulting in higher C═C intensity for the 4 C formation step. Other C 1s peaks emerge for the SEI coated samples which indicate the formation C─O (286.4 eV), C═O (288.1 eV), O─C═O (290.4 eV), C─F (291.4 eV) within the SEI because of the complex reduction reactions involving ethylene carbonate (EC), dimethyl carbonate (DMC) and LiPF_6_ in the electrolyte. A similar reduction in intensity is confirmed for the Ti 2p signal (Figure 6b) where intensity corresponding to Ti─C (460 eV) and Ti─O (465 eV) bonding are clearly detected with the control and the electrodes with SEI formed at 4C while it is completely obscured for electrodes where the SEI was formed at 0.1C. Titanium is only present in the electrode bulk and not in the formed SEI layer which again indicates that the SEI formed at 4C is significantly thinner than the SEI formed at 0.1C. The Li 1s and O 1s signals (Figure 6c,d) indicate SEI with a similar composition but more Li and O due to the thicker SEI formed in the 0.1 C case. All these results point to a thinner and more Li‐ion conducting SEI formed for the 4C formed electrodes which was enabled by the higher electronic conductivity of the covalently joined TiC–graphite.

Compared to other recent works that demonstrate high cycle‐life under XFC, we do so in a half‐cell configuration that cycles the graphite anode through an entire lithiation/delithiation cycle using a significant excess of cathode capacity (4 mAh cm^−2^ NMC counter electrodes) to ensure we are limited by the anode (1 mAh cm^−2^). On the other hand, recent examples feature negative to positive electrode ratios (N:P) between 1.2 and 1.05.^[^ ^35^, ^75^ ^]^ In these cases, there is only enough lithium to cycle between 79 and 95%, at the most, of the actual graphite capacity. To these figures, we must also subtract the lithium lost to SEI formation that varies between 87.2% to 94.5% depending on the carbon‐coating of the particles. These considerations result in effective graphite electrode cycling capacities that vary widely from 95.2% × 94.5%  = 90.0% to as low as 78.9% × 87.2% =  68.8%.^[^ ^76^ ^]^ This practice imposes a lithium‐starved SEI formation, and a relatively shallow depth of charge when one considers the anode alone. It precludes the fundamental study of the inherent performance of the full intercalation kinetics of the graphite anode. As far as we know no study where all the capacity of graphite electrodes with loading greater than 1 mAh cm^−2^ has been conducted leaving a fundamental research gap which we addressed in our work. This is especially important when it comes to the studying of further manipulation of SEI formation beyond the accepted slow formation step at 0.1C. Electrode with high loading (>3 mAh cm^−2^) were also tested with results presented in Figure S9 (Supporting Information). However, control electrodes purchased from NEI were not able to cycle reliably under XFC cycling at room temperature and with the electrolyte used in this study. It is likely that the porosity of these commercially available electrodes is not optimized for fast charging, whereas electrodes with capacity slightly over 1 mAh cm^−2^ do not suffer from this problem and allowed for this comparison study to be conducted.

## 3 Conclusion

This application of the novel covalently joined electrode architecture to graphite anodes for Li‐ion batteries demonstrates key advantages in charge conductivity, electronic and ionic, as well as significant improvement in cycle life and longevity compared to commercially made electrodes using the traditional polymer and conductive additive architecture. This study demonstrates that chemically joining active material to the current collector provides electrodes with a progressive mechanical failure mode that allows for longer operation at full capacity compared to the traditional electrode with polymer binder that tend to fail catastrophically through the severance of electrical pathways through delamination and film disintegration. With the fast‐development of the electric revolution of transportation set to take full effect in the middle of the 2030′s, Li‐ion batteries are called upon to power most electric vehicles. This role will put large pressure on their ability to last under many forms of abuse including fast charging at lower temperature as required for mass adoption according to the US DoE. Covalently joined architecture provides a strong answer to mitigating electrode degradation and impedance growth allowing electrodes to be charged without suffering capacity loss over hundreds of cycles lasting the life of electric vehicles. Applications for covalently joined electrode architectures are not limited to Li‐ion graphite anodes as such architecture can be implemented and provide similar benefits indifferently to all other electrodes for electrochemical energy storage including Na‐ion and lithium sulfur battery, supercapacitor as well as the upcoming solid state battery applications. The presented implementation using TiH_2_ as precursor for bonding agent for carbon (or carbon coated) active materials is but one declination of the novel electrode building method. Further research is necessary to develop and implement new joining agents such as silicon nanoparticles or ternary carbide precursor with lower decomposition temperature and improving the distribution of the covalently bound network to make the technology more scalable and further improve performance. The uniformity of the covalent should also be improved most likely by replacing the solid metal precursor with a liquid precursor that can conform to the surface of particles prior to bonding with them. Finally, reducing bonding reaction temperatures and eliminating hydrogen degassing will make the process less energy intensive and safer.

## 4 Experimental Section

### Materials

Mesocarbon microbeads (MCMB) graphite powder was purchased from Nanografi. Artificial graphite powders and pre‐made graphite (1 mAh cm^−2^) tape and NMC‐811 (4 mAh cm^−2^) were procured from NEI corporation. Titanium hydride micro powders were bought from Sigma‐Aldrich, while nanosized titanium hydride powder was sourced from Nano‐Research. The electrolyte, a 1 molar lithium hexafluorophosphate in 1:1 (by volume) mixture of ethylene carbonate (EC) and dimethyl carbonate (DMC), was purchased from Sigma‐Aldrich. The polymer binder, polyacrylic acid was purchased as an aqueous dispersion also from Sigma‐Aldrich. 0.032 mm thick titanium foil was purchased from Alfa Aesar. All of the chemicals were used as received with no further treatment. All of these chemicals, expect for the titanium hydride micro powder, the polyacrylic dispersion and the titanium foil, were stored in an argon‐filled glovebox.

### Fabrication of Pellets for XRD Characterization and Mechanical Testing

Pellets are made from sintered MCMB graphite and TiH_2_ powders with molar ratio ranging from 1:1 to 4:1. Sintering was conducted in a hot press under argon at temperatures of 800 and 1000 °C for 20 min with a ramp of 10 °C min^−1^ under 34.7 MPa.

For XRD characterization, the pellets were crushed to a fine powder using pestle and mortar. Bulk chemical characterization of the composite materials was conducted on a Rigaku Miniflex II X‐Ray diffractometer with a copper target.

For mechanical bending characterization, pellets were placed under water and cut into test samples using a water jet. Finally, the standardized 1.5 mm  ×  5 mm  × 25 mm size was achieved by manual grinding.

### Sheet Resistance Characterization

Four‐point probe conductivity measurements were performed along the top surface of films cast on an alumina substrate that was electrically insulating. These films were not compacted due to limitations in the mechanical properties of the alumina support.

### Tear Test

Tear test samples were prepared identically to the covalently joined electrode except that they were prepared as a sandwich with metal foils on both side of the active materials. Metals foils are copper or titanium. To execute the test one side of the sandwich is secured using super glue to a glass slide and the other side is similarly secured to a 5 mm diameter stub equipped with a pulling string. The stub is tied to a load cell that is continuously pulled until the sandwich fails and separates perpendicularly to its layers.

### Bending Test

Standardized 1.5 mm  ×  5 mm  × 25 mm test beams made from the graphite‐TiC composite are tested for bending strength following the ASTM C1161‐18 test standard for flexural strength of advanced ceramics at ambient temperature using a custom three‐point configuration bench (Figure 3a) fabricated in‐house.

### Electron Microscopy

The particle morphology and cross‐section images were obtained with scanning electron microscopy (SEM) (Zeiss LEO 1530 & Tescan VEGA TS‐5130) under high vacuum and accelerating voltage ranging from 5 to 20 kV. Samples were affixed onto aluminum studs using carbon tape and imaged without any additional conductive coatings. Energy dispersive X‐ray diffraction (EDS) was conducted on Zeiss LEO 1530 under an accelerating voltage of 20 kV.

### High‐Resolution Transmission Electron Microscopy

To determine the thickness of the SEI, transmission electron microscopy was performed at the University of Waterloo's QNFCF facility using a JEOL JEM‐F200 S/TEM at 200 kV. Characterization of the crystalline structure of the graphite–titanium carbide composite material was obtained with high‐resolution transmission electron microscopy at the Canadian Center for Electron Microscopy facility at McMaster University, Canada using a ThermoFisher Scientific Spectra Ultra, double‐corrected HRTEM/STEM operated at 300 kV. The powder sample was affixed to a carbon film supported on a copper grid.

Characterization of the chemical makeup of the solid electrolyte interphase layers was via X‐ray photoelectron spectroscopy (XPS) characterization conducted on a ThermoFisher Scientific Nexsa G2 Surface System with a monochromated Al Kα.

### Electrode Fabrication

Covalently joined electrodes were prepared by adding NEI graphite and titanium hydride (TiH_2_) nanoparticles to an aqueous dispersion of polyacrylic acid (PAA) in the following proportions: 90 parts graphite, 5 parts TiH_2_ and 50 parts PAA. The slurry was casted on a titanium foil and dried overnight at 75 °C. Dried slurry and titanium foil were then punched out using a precision cutter and weighted to determine the active material (graphite) loading. The punched‐out disks were then cold pressed under 88.5 MPa followed by sintering under argon at 800 °C for 20 min with a ramp of 10 °C min from room temperature.

### Cell Assembly

Three‐electrode ½ in. Swagelok cells (Figure S1, Supporting Information) were exclusively used as half‐cells. These cells were assembled in an argon‐filled glovebox. Working and counter electrodes were punched on a precision cutter from MTI with a 12 mm diameter. These cells used a lithium foil cut to ½ inch diameter and pressed against the current collector in the top position of the Swagelok cell. The lithium foils were brushed to remove any trace of oxide layer on its surface prior to assembly. Working electrodes were either the lab‐made covalently joined architecture using graphite powder purchased from NEI, or the graphite electrodes made by NEI using the same graphite powder. Loading of the NEI‐made electrodes was 1 mAh cm^−2^ and loading of the covalently joined architecture was between 1 and 1.2 mAh cm^−2^. Counter electrodes were NMC‐811 electrodes made by NEI with 4 mAh cm^−2^ loading. Working, counter, and reference electrodes were all separated by glass fiber separator punched at ½ in. diameter and purchased from Whatman. The separators were soaked with 200 µl of 1 m LiPF6 in (1:1) by volume EC:DMC purchased from Sigma Aldrich.

### Battery Cycling

The accelerating aging test was performed on the assembled Swagelok cells on a Biologic VSP‐300 potentiostat and Arbin instrument cycler. Lithiation (charge) of the graphite electrodes was done using a constant current‐constant voltage routine with a 15 min maximum total time. Constant current was adjusted at 3.2C with respect to the total specific capacity of 370 mAh g^−1^ measured by the manufacturer NEI during their 0.1C charge–discharge cycling. Constant voltage was set at 0.02 or 0.06 V versus the Li/Li^+^ reference electrode (cf. Figure S4, Supporting Information). Delithiation (discharge) was performed through a constant current corresponding to 1C based on the 370 mAh g^−1^ capacity. This delithiation current is maintained until the potential of the graphite reaches 2 V versus Li/Li^+^ refence electrode. All electrochemical impedance spectroscopy characterization was done at 2 V versus Li/Li^+^. These graphite electrodes were tested at room temperature (23 ± 2 °C) using three‐electrode Swagelok cells against NMC‐811 electrodes purchased from NEI corporation used as counter electrodes and lithium metal used as reference electrode (cf. Figure S1, Supporting Information). Commercial graphite electrodes purchased from NEI with 1 mAh cm^−2^ loading. Covalently joined graphite electrodes were produced in‐house with capacity ranging from 1 to 1.2 mAh cm^−2^ using the same graphite powder from NEI as the commercial electrodes.

## Conflict of Interest

This work is protected by an international patent application filed by the Y.R. and M.A.P. through the University of Waterloo commercialization arm (WatCo).

## Supporting information



Supporting Information

## Data Availability

The data that support the findings of this study are available from the corresponding author upon reasonable request.
