# Supplementary material for: Covalent Carbide Interconnects Enable Robust Interfaces and Thin SEI for Graphite Anode Stability under Extreme Fast Charging
Source: Adv Sci (Weinh). 2024 Oct 23;11(46):2408277. doi: 10.1002/advs.202408277 (PMC11633473; doi:10.1002/advs.202408277)
Supplement: Supplementary file 1 — Supporting Information [file ADVS-11-2408277-s001.docx]

**Supplementary information**

**Covalent Carbide Interconnects Enable Robust Interfaces and Thin SEI for Graphite Anode Stability under Extreme Fast Charging**

Yverick Rangom,* Oleksii Sherepenko, Ahad Shafiee, Alek Cholewinski, Kiran Gundegowda Kalligowdanadoddi, Bersu Bastug Azer, Parisa Jafarzadeh, Boxin Zhao, Elliot Biro, Holger Kleinke, Michael A. Pope*

Y. Rangom, A. Cholewinski, K. Gundegowda Kalligowdanadoddi, B. Zhao, M. A. Pope

Department of Chemical Engineering

University of Waterloo

Waterloo, Canada

E-mail: [yverick.pascal.rangom@uwaterloo.ca](mailto:yverick.pascal.rangom@uwaterloo.ca); [michael.pope@uwaterloo.ca](mailto:michael.pope@uwaterloo.ca)

O. Sherepenko, A. Shafiee, E. Biro

Department of Mechanical and Mechatronics Engineering

University of Waterloo

Waterloo, Canada

P. Jafarzadeh, H. Kleinke

Department of Chemistry

University of Waterloo

Waterloo, Canada

**Figure S1.** Schematic of three-electrode half-cell for testing graphite anodes.

All graphite electrodes were tested using three-electrode Swagelok cells with a NMC-811 counter electrode with 4 mAh/cm^2^ capacity and lithium metal reference electrode.

**Figure S2.** X-ray diffraction analysis. **(a)** Comparison of ketjen black and TiH_2_ 4:1 mixture powders conditioned differently before sintering at 800 °C. **(b)** Comparison of ketjen black and TiH_2_ 4:1 mixture pressed powders sintered at different temperatures with XRD profile of purchased titanium carbide powder. **(c)** Comparison of ketjen black and TiH_2_ 4:1 mixture pressed powders sintered for 10 minutes and for 1 hour. **(d)** XRD characterisation of PAA film sintered under argon on titanium foil at 1000 °C under 34.7 MPa of pressure (red). **(e)** XRD characterisation of NEI graphite particles sintered under argon on a titanium foil at 1000 °C under 34.7 MPa of pressure (red).

Samples of 4:1 molar ratio of ketjen black to TiH_2_ mixture were sintered in a mullite tube furnace and after different conditioning. **Figure S2a** shows that cold pressing of the powders is required for titanium carbide (TiC) to form whereas ball milling priming the reaction through ball milling is insufficient on its own to sustain TiC formation. **Figure S2b** shows that TiC formation can occur at temperature as low as 400 °C. **Figure S2c** demonstrate the rapidity of the reaction occurring completely after merely 10 minutes at 600 °C.

**Figures S2 d&e** show the XRD characterisation of PAA film and graphite respectively pressed against titanium under 34.7 MPa of pressure and sintered at 1000 °C. Comparing the by-products to titanium carbide and titanium metal it is clear that carbonized PAA forms TiC under these conditions while graphite does not to react. The graphite (004) peak observed comes from graphite transfer from the graphite foil (grafoil) liner used in this hot press experiment as it sticked to this particular sample. A graphite die and liner are required to maintain 34.7 MPa pressure at 1000 °C. Graphite transfers from grafoil was not an issue in any other experiment presented here.

**Figure S3.** Thermogravimetric analysis of polyacrylic acid under nitrogen atmosphere.

Covalently joined electrodes used a polyacrylic acid (PAA) based slurry that was subsequently sintered at 800 °C under argon. Thermogravimetric analysis of PAA under nitrogen flow shows that that the carbon residue weighs 10% of the original weight.

**Figure S4.** Electrochemical characterisation of cycle life of 1 mAh/cm^2^ graphite anode half-cells with 4 mAh/cm^2^ NMC-811 counter electrodes – 15-min charge (3.2C constant current, and constant voltage at either 0.02 or 0.06 V vs. Li/Li^+^) and 1C discharge (a) for traditional slurry architecture electrodes **(b)** for covalently-joined architecture electrodes.

The extreme fast charging protocol used in this study utilizes a constant current at 3.2C followed by a constant voltage at either 0.02 or 0.06 V vs. Li/Li^+^ followed by a constant current discharge at 1C. A higher constant voltage is designed to help mitigate metal plating. This protocol is applied to covalently joined graphite electrodes after solid electrolyte interphase is formed under 0.1C or 4C current density. In both **Figures S4 a** & **b**, cells cycled with a constant voltage charge at 0.06V vs. Li/Li^+^ reach a consistently lower state of charge. However, in **Figures S4 a** & **b** the capacity loss is identical showing that, for both the commercial graphite electrode and the covalently joined electrode there is no difference in metal plating mitigation when charging is stopped at 0.02 or 0.06 V vs. Li/Li^+^. The commercial electrode displays the same capacity decay regardless of the charge voltage limit indicating that this decay (cf. **Figure S4a**). However, the covalently joined electrodes displays no capacity loss regardless of constant voltage limit indicating that no metal plating is occurring on these electrodes even when the charge voltage limit is 0.02V vs. Li/Li^+^ (cf. **Figure S4b**).

**Figure S5.** Schematic of equivalent circuit of test cells with graphite electrode at 2V vs. Li/Li^+^.

This equivalent circuit describe the electrochemical impedance spectroscopy (EIS) behaviour of our graphite-NMC cells when tested with a graphite electrode bias voltage of 2V vs. Li/Li^+^. The model contains:

- series resistance R1 models the ionic resistance of the electrolyte solvent
- constant phase element (CPE2) in parallel with resistance R2 model the combined electronic interface of the cell
- constant phase element (CPE3) in parallel with resistance R3 model the combined ionic interface of the cell
- a Warburg element models the capacitance and diffusion characteristics of ions in the electrolyte and through SEI layer

**Figure S6.** Electrochemical characterisation of 1 mAh/cm^2^ graphite anode half-cells with 4 mAh/cm^2^ NMC-811 counter electrodes – a.b.c. 15-min charge (3.2C constant current, 0.02 V vs. Li/Li^+^ constant voltage) and 1C discharge. Cycle life comparison of covalently joined electrode (black dotted line) to sintered electrodes with **(a)** titanium current collector without TiH_2_ precursor (cell 1); **(b)** copper current collector with TiH_2_ precursor (cell 2); **(c)** copper current collector without TiH_2_ precursor (cell 3). **d.e.f.** Electrochemical Impedance Spectroscopy of 1 mAh/cm^2^ graphite anodes after increasing number of cycles **(d)** for cell 1; **(e)** for cell 2; **(f)** and for cell 3.

For cells 1 and 3, the TiH_2_ precursor has been removed from the slurry leaving only 55 parts PAA and 90 parts graphite. This slurry is then pasted on titanium (cell 1) or copper (cell 3) and sintered. Cell 2 is made with the same slurry as in the main text containing 5 parts TiH_2_, 50 parts PAA and 90 parts graphite but it is sintered on a copper current collector. Therefore, the architecture of cell 1 includes confirmed TiC chemical bonds (cf. **Figure S3d**) between film and titanium current collector. While the chemistry of cell 2 can only form a chemical bond between current collector and film via alloying of copper and titanium from the TiH_2_ particles. Finally, the architecture of cell 3 cannot form any chemical bond between the current collector and film as copper does not form a carbide.

**Figure 5a** shows the loss of capacity for cell 1 is nearly identical to the sintered electrode with TiH_2_ not withstanding the disturbances highlighted by asterisks. These disturbances occurred when the electrical contacts of this particular cell were physically bumped during the two months of cycling. The sintered cell using TiH_2_ precursor and a copper current collector displayed a larger loss of capacity compared to the sintered cells using a titanium current collector (**Figure S6b**) while the sintered cell using a copper current collector but no TiH_2_ precursor exhibited a much larger capacity loss over 800 cycles (**Figure S6c**).

Electrical impedances of these cells are tracked by EIS characterisation conducted with EIS test is conducted with a DC offset of 2V vs. Li/Li^+^ to prevent intercalation in the graphite and favour a supercapacitor-like charge storage based on surface adsorption. In these conditions, the size of the high frequency semi-circle on the Nyquist plot indicates the electrical impedance of a given cell. These Nyquist plots are displayed in **Figures S6 d, e, and f**. We observe that the high frequency semi-circle for the cell using copper current collector and no TiH_2_ precursor is significantly larger than the other two cells denoting an electrical impedance that is also naturally significantly larger for this cell. It is significant to note that copper does not form any form of chemical bond. Therefore, there is necessarily no chemical bond ensuring neither a continuous electrically conductive media between current collector and film nor mechanical integrity preventing delamination for this cell. This result combined with the lower cycle life for this cell confirms the

We observe that none of these sintered electrodes exhibit any significant growth of the high-frequency semi-circle, as shown in **Figures S6 d, e, and f**, indicating little fundamental degradation of electrical contacts, whether with the current collector or in-between particles. However, one notable difference between these sintered electrodes is the larger semi-circle size exhibited by cell 3 indicating that the chemistry of electrical contact of this cell is fundamentally different that cells 1 and 2, likely indicating the electrical difference between continuous electrical medium between current collector and film (cell 1) versus electrical conductivity by contact (cell 3).

Finally, the height of the tail on the Nyquist plot remains constant after 800 cycles for cell 3 (**Figure S6f**) denoting a constant capacitance therefore no loss of active surface area and by extension of active material. Therefore, the loss of capacity for this last electrode is likely due other phenomena than “dead” (separated from the rest of the electrode) active material. We conclude that progressive degradation of the physical contact between current collector and film by delamination and SEI build-up is the main cause for the poor cycle life.

**Figure S7.** Electrochemical characterisation of 1 mAh/cm^2^ graphite anode half-cells with 4 mAh/cm^2^ NMC-811 counter electrodes – 15-min charge (3.2C constant current, and 0.06 V vs. Li/Li^+^) and 1C discharge with solid electrolyte interphase (SEI) formed at 0.1 and 4C during the 1^st^ cycle. (a) Cycle life characterisation for traditional slurry architecture electrodes. (b) Cycle life characterisation for covalently joined architecture electrodes. (c) SEI formation profiles. (d) Comparison of coulombic efficiency and irreversible capacities for SEI made at 0.1 and 4C on covalently joined architecture electrodes.

SEI formation rate has no effect on the cycle life of the commercial electrode as shown in **Figure S7a**. Capacity loss is similar for electrodes with SEI formed under 0.1 and 0.4C current densities. The electrode with SEI formed at 0.1C displayed severe metal plating during its first cycle with a specific capacity of nearly 700 mAh/g that is well above the 340 mAh/g theoretical capacity. On the other end, Figure S5b shows that the SEI formation rate has a dramatic effect on cycle life for covalently joined electrodes. Cells with SEI formed under the traditional 0.1C showing a constant capacity decay, whereas the cells with SEI formed at 4C show no capacity fade (cf. **Figure S7b**). It is safe to conclude that SEI formation under high current rate on an electrode with superior electrical conductivity is necessary to ensure long cycle life. **Figure S7c** shows identical SEI formation profile for both commercial and covalently joined electrode when the formation step is conducted at 0.1C demonstrating that the new architecture doesn’t introduce a penalty in the amount of SEI formed compared to traditional electrodes. This indicates that despite being more conductive the covalently joined electrode does not noticeably produce more SEI than the commercial electrode. The commercial electrode seems to have a higher capacity when SEI is formed at 4C. It is likely that the additional capacity is due to metal plating. **Figure S7d** shows another advantage of forming SEI at higher rate. The irreversible capacity is about 43% when SEI is formed at 4C (80 mAh/g) versus at 0.1C (140 mAh/g).

**Comparison with reports on full cell:**

Several studies have explored strategies to enhance the fast-charging capabilities of graphite anodes. Sun et al. demonstrated promising results with surface-modified graphite, achieving 86.6% capacity retention after 1500 cycles at a high charge rate (6C) but a slower discharge rate (0.2C).^[1]^ This highlights the inherent trade-off between fast charging and cycling stability in conventional graphite anodes. Our work presents a novel approach utilizing electrodes with a covalent architecture. These electrodes exhibit superior cycling stability, retaining 98% of its initial capacity after 800 cycles even at a significantly faster discharge rate (1C).

Chen et al. investigated fast charging with Graphite/hard carbon (HC) hybrid anodes, achieving 88% capacity retention after 500 cycles at a 4C-1C charge-discharge protocol.^[2]^ Their improved performance is attributed to better current distribution and reduced lithium plating during fast charging. In this work, we introduce a modified graphite anode featuring covalent carbide interconnects formed by sintering graphite with TiH_2_. This modification demonstrably enhances both electronic and ionic conductivity, leading to negligible capacity fade over 800 cycles. This improved performance suggests the potential of covalent architecture electrodes for high-performance LIB applications demanding fast charging capabilities.

Additionally, Chen et al. reported a highly ordered laser-patterned graphite electrode achieving fast charging at a 4C charge and 0.5C discharge rate, while retaining 91% capacity after 600 cycles.^[3]^ This design utilizes vertical pores as diffusion paths for Li-ion transport, reducing concentration polarization during fast charging. Our novel approach offers a promising alternative, with the covalent architecture promoting both electronic and ionic conductivity while maintaining excellent fast-charging and cycling stability.

**Figure S8.** High-resolution TEM of SEI layer formed under (a) 0.1C and (b) 4C current densities.

SEI layers were formed on the surface of covalently joined graphite particles under 0.1C and 4C current densities.

**Figure. S9.** Electrochemical characterisation of >3 mAh/cm^2^ graphite anode half-cells with 4 mAh/cm^2^ NMC-811 counter electrodes – 15-min charge (3.2C constant current, and 0.02 V vs. Li/Li^+^) and 1C discharge. Specific capacity over 800 cycles are shown in blue (solid line) and coulombic efficiency is shown in orange (dotted line) for (a) NEI purchased control graphite electrode, (b) Covalently joined architecture graphite electrode.

Graphite anodes with loading over 3 mAh/cm^2^ were tested following the same procedure XFC used with the 1 mAh/cm^2^: lithiation of graphite anodes at 3.2C constant current followed by constant voltage at 0.02V vs. Li/Li^+^ with a combined time of 15 minutes, and delithiation at 1C until the graphite anodes reaches 2V vs. Li/Li^+^. Results for the control electrode purchased from NEI and for a covalently joined graphite anodes are shown in **Figure S8 a&b** respectively. **Figure S8a** shows a rapid decrease in specific capacity to below 50 mAh/g after 800 cycles while the covalently joined electrodes registered about 150 mAh/g after 800 cycles (**Figure S8b**). More importantly the control electrode cannot maintain a ~ 100% coulombic efficiency demonstrating inadequacy of the high loading electrodes bought from NEI while the covalently joined electrode is able to maintain ~100% coulombic efficiency over the entire 800 cycles.

**Figure S10.** Charge-discharge curves of covalently joined pure TiC electrodes cycled at different current densities (37, 370 and 1184 mA/g) from 0.02 to 2V vs. Li/Li^+^.

Pure TiC electrodes were produced by sintering 1-to-1 mixture of TiH_2_ and carbon particles at 800 °C under argon atmosphere. This electrode demonstrates the specific capacity of the TiC material used as joining material in this study. Specific capacities of 120, 20.5, and 7.95 mAh/g are obtained at 37, 370, and 1184 mA/g respectively. These current densities correspond to used to ones used test the graphite electrodes at 0.1, 1, and 3.2C respectively in the main study.

During cycling of the graphite electrodes, current densities of 3.2C on lithiation and 1C on delithiation are employed. It follows that the additional capacity provided by 5 wt% TiC content in the graphite covalently joined electrode when cycled at 3.2C is at most 1 mAh/g (calculated using 20.5 mAh/g at 1C) out of the total 280 mAh/g measured representing about 0.4% of the total measured capacity. Therefore, this additional capacity from lithium stored in the TiC material is negligeable.

**Table S1.** Evolution of the electrical impedance of graphite electrodes after 1, 50 and 800 cycles

|  | Cycle number | Electrical impedance (R2)  Ω.cm^2^ |
| --- | --- | --- |
| Commercial NEI control architecture | 1 | 2.9 |
|  | 50 | 9.2 |
|  | 800 | 61.4 |
| Sintered architecture with TiH_2_ on titanium (“TiC joined architecture”) | 1 | 3.3 |
|  | 50 | 3.2 |
|  | 800 | 4.3 |
| Sintered architecture without TiH_2_ on titanium | 1 | 1.8 |
|  | 50 | 1.6 |
|  | 800 | 2.2 |
| Sintered architecture with TiH_2_ on copper | 1 | 4.8 |
|  | 50 | 6.9 |
|  | 800 | 7.9 |
| Sintered architecture without TiH_2_ on copper | 1 | 7.3 |
|  | 50 | 7.2 |
|  | 800 | 8.9 |
